# Prolonged mosquitocidal activity of *Siparuna guianensis* essential oil encapsulated in chitosan nanoparticles

**DOI:** 10.1371/journal.pntd.0007624

**Published:** 2019-08-09

**Authors:** Taciano P. Ferreira, Khalid Haddi, Roberto F. T. Corrêa, Viviana L. B. Zapata, Tathyana B. Piau, Luis F. N. Souza, Swel-Marks G. Santos, Eugenio E. Oliveira, Luis O. V. Jumbo, Bergmann M. Ribeiro, Cesar K. Grisolia, Rodrigo R. Fidelis, Ana M. S. Maia, Raimundo W. S. Aguiar

**Affiliations:** 1 Postgraduate Program of the Legal Amazon Biodiversity and Biotechnology Network - Rede Bionorte, Universidade Federal do Tocantins, Palmas, Tocantins, Brazil; 2 Departamento de Química Ambiental, Universidade Federal do Tocantins, Gurupi, Tocantins, Brazil; 3 Departamento de Entomologia, Universidade Federal de Viçosa, Viçosa, Minas Gerais, Brazil; 4 Departamento de Biologia Celular, Universidade de Brasília, Brasília, Distrito Federal, Brazil; 5 Departamento de Genética e Morfologia, Instituto de Ciências Biológicas, Universidade de Brasília, Brasília, Distrito Federal, Brazil; 6 Departamento de Engenharia de Bioprocessos e Biotecnologia, Universidade Federal do Tocantins, Gurupi, Tocantins, Brazil; 7 Departamento de Produção Vegetal, Universidade Federal do Tocantins, Gurupi, Tocantins, Brazil; Faculty of Science, Mahidol University, THAILAND

## Abstract

**Background:**

The use of synthetic insecticides is one of the most common strategies for controlling disease vectors such as mosquitos. However, their overuse can result in serious risks to human health, to the environment, as well as to the selection of insecticidal resistant insect strains. The development of efficient and eco-friendly insect control is urgent, and essential oils have been presented as potential alternatives to synthetic insecticides. Moreover, nanoencapsulation techniques can enhance their efficiency by protecting from degradation and providing a controlled release rate.

**Results:**

We assessed the potential of chitosan nanoparticles in encapsulating *Siparuna guianensis* essential oil, and maintaining its efficiency and prolonging its activity for the control of *Aedes aegypti* larvae. The encapsulation was characterized by scanning electron microscopy (SEM), Fourier-transform infrared spectroscopy (FTIR), and thermogravimetric analysis (TGA), with an encapsulation efficiency ranging from 84.8% to 88.0%. Toxicity studies have demonstrated efficacy against mosquito larvae over 50% for 19 days with 100% mortality during the first week. This persistent action is presumably due to the enhanced contact and slow and maintained release conferred by chitosan nanoparticles. Furthermore, the exposure of aquatic non-target organisms (e.g. embryos and small adult fishes) revealed adequate selectivity of these nanoparticles.

**Conclusions:**

The encapsulation of *S*. *guianensis* essential oil in chitosan nanoparticles showed promising potential as a larvicide control alternative and should be considered within strategies for fighting *Ae*. *aegypti*.

## Author summary

Numerous outbreaks of infectious diseases such as dengue, zika, and chikungunya in tropical countries have occurred where the mosquito *Aedes aegypti* is the transmitting vector. In Brazil, these infections are responsible for deaths and severe sequelae. Thus, many efforts have been made by governmental and research groups to control these outbreaks. However, complete success in this control has so far remained unachieved. Parallel to the need to develop new technologies that contribute to the control of insects that transmit diseases, there is a growing societal awareness regarding the risks associated with the use of synthetic insecticides, which has led to a search for natural alternatives such as essential oils from plants. Thus, our group conducted experiments to evaluate the application of nanotechnology in obtaining an efficient prolonged release system to combat *Ae*. *aegypti* larvae using the essential oil of a plant native to the Cerrado and Amazonian forests. These results demonstrate that using a simple and easily scalable encapsulation technique; it is possible to keep the low toxicity against non-target organism and prolong the activity of an essential oil in water and maintain larval mortality at a significant level for more than a week with a single application.

## Introduction

The *Aedes aegypti* Linnaeus, 1762 (Diptera: Culicidae) mosquito is a vector for yellow fever, dengue, Zika, and chikungunya viruses, and can be found throughout the American continents [1–3], in Africa, and Asia [4]. Its control is mainly through insecticides; usually, pyrethroids are used against adults and temephos against larvae [5]. However, *Ae*. *aegypti* populations from various regions of the planet have been shown to be resistant to these and other insecticides [6–10]. The microbial larvicide *Bacillus thuringiensis* var *israelensis* (Bti) is successfully used against mosquito larvae worldwide since decades. Bti principal disadvantage in mosquito control programs is the lack of evidence that recommends its use as single agent for the vector control [11].

In this context, essential oils could be an alternative to conventional insecticides. They are volatile oils of complex composition produced by plants as secondary metabolites, and due to their complex compositions, they may contribute to the delay of the emergence of resistant insects [12, 13]. Research on *Siparuna guianensis* essential oil, also known as negramina or capitiú in certain regions of Brazil, have demonstrated its potential in vector and pest insect control, such as *Ae*. *aegypti* and *Culex quinquefasciatus* Say, 1826 (Diptera: Culicidae) [14], and wax moths *Achroia grisella* and *Galleria mellonella* [15]. However, as observed for other essential oils, their volatility and easy oxidation can compromise their persistence in the medium, requiring a high number of applications, and their immiscibility in water makes it difficult to use as larvicides [12, 16].

A good approach to preserve essential oil properties and improve its dispersibility in water is encapsulation in polymer nanoparticles [16–18]. Among the polymers used for encapsulation, chitosan stands out due to its biological properties, such as non-toxicity, biocompatibility, biodegradability, and its production from renewable sources, besides being a polycation, which contributes to its cellular internalization [19–21]. Some examples of essential oils that have been encapsulated in chitosan nanoparticles include oregano [22], cinnamon [23], lime [24], and summer savory [25] essential oils.

Several studies demonstrate the encapsulation of essential oils in chitosan nanoparticles, and the physicochemical properties of these nanoparticles have been extensively studied. However, few studies have investigated their toxic action to mosquito larvae in an aqueous medium and their safety in relation to other non-target species. Only two articles describe chitosan-based nanoparticles with *Lippia sidoides* essential oil against *Ae*. *aegypti* larvae Abreu, Oliveira [26], [27], and only one study teste chitosan encapsulated oils of *Geranium maculatum* and *Citrus bergamia* against larvae of *Cx*. *pipiens pipiens* [28]. In these studies, mortality greater than 80% was observed in the first four days.

For use as a larvicide, it is crucial that the insecticide remains for enough time in the aqueous medium where larvae develop to have an effective action. Thus, this study aimed to verify the duration which chitosan nanoparticles crosslinked with glutaraldehyde maintain the residual larvicidal activity of *S*. *guianensis* essential oil in an aqueous medium. Glutaraldehyde was chosen as a crosslinking agent because it was found that chitosan nanoparticles crosslinked with it had enhanced stability in an aqueous medium [29]. We characterized the physicochemical properties of the loaded nanoparticles and assessed their toxicity, residual effects and selectivity as an alternative control strategy of *Ae*. *aegypti* larvae.

## Methods

### Materials

Chitosan was purchased from Sigma-Aldrich (Brazil). Its viscosity-average molecular weight was 4.9 x 10^4^ g mol^-1^, estimated at 25°C in 0.3 M acetic acid/0.2 M sodium acetate. Its degree of deacetylation was 76.5%, determined by proton nuclear magnetic resonance ^1^H NMR, according to procedures described elsewhere Lima Vidal, Pereira Fagundes [30]. Glutaraldehyde was obtained from Sigma-Aldrich. Polysorbate 80 U.S.P. grade was purchased from Synth (Brazil). All the reagents were used as received.

### Collection of *S*. *guianensis* leaves

*S*. *guianensis* leaves were collected in Gurupi (11°43'45" latitude S, 49°04'07" longitude W), Tocantins state, Brazil. The research was registered and approved by the Genetic Patrimony—National Council of Scientific and Technological Development, CNPq, n° 010580/2013-1. Experts confirmed taxonomic identification at the herbarium of the Federal University of Tocantins (Campus Porto Nacional), where samples were deposited with reference number 10.496.

### Extraction and identification of the *S*. *guianensis* essential oil

The *S*. *guianensis* leaves were subjected to hydro-distillation for 2 h in a Clevenger apparatus according to the procedure described by Aguiar, dos Santos [14] with an average yield of 0.81 g of essential oil per cycle. The extracted oil sample was stored in sealed vials at -80°C. The chemical composition was determined by gas chromatography-flame ionization detector (GC-FID) using a Chemito 8510 GC instrument (Chemito Technologies Ltd, Mumbai, India Pvt.) and gas chromatography-mass spectrometry (GC-MS) analysis with a DSQ MS screen was performed using Thermo Electron Corporation equipment, Waltham, MA, USA, as previously described [14].

### Insects

Third instar *Ae*. *aegypti* larvae were obtained from cultures maintained in the Integrated Pest Management (IPM) laboratory- Universidade Federal do Tocantins as described elsewhere [14].

### Preparation of chitosan–essential oil nanoparticles

Chitosan nanoparticles containing essential oil were prepared according to a procedure modified from those previously reported in the literature [31, 32]. A solution of 0.5% (w/v) chitosan was prepared by dissolving the chitosan in 2% acetic acid solution, under magnetic stirring overnight at room temperature. Polysorbate 80 was added to the chitosan solution with constant stirring at 45°C for 2 h to obtain a surfactant concentration of 1%. The solution was cooled to ambient temperature, and different amounts of essential oil were added dropwise into the chitosan solution by stirring vigorously to obtain oil-in-water emulsions. The oil-in-water emulsions were gradually dropped into a glutaraldehyde solution (5%, pH 5) under constant stirring at room temperature. The resultant nanosuspensions were freeze-dried and stored in a desiccator. The nanoparticles without oil were named as CN and those with oil as CO followed by the chitosan:essential oil proportions as shown in Table 1. Chitosan nanoparticles without essential oil were obtained following the same procedure.

**Table 1:** Nanoparticles names and chitosan:Essential oil proportions used in preparation.

Sample code	Chitosan	Essential oil
**CN**	1.0	0.0
**CO2:1**	1.0	0.5
**CO1:1**	1.0	1.0
**CO1:2**	1.0	2.0

### Characterization of nanoparticles

#### Structural characterization

Fourier-transform infrared spectroscopy (FTIR) spectra were obtained with an IRAffinity-1 Fourier transform infrared spectrometer from Shimadzu, coupled to an Attenuated Total Reflectance (ATR) MIRacle module with an ZnSe prism, manufactured by PIKE technologies, using 32 scans at a resolution of 4 cm^−1^, from 4000 to 700 cm^−1^.

#### Thermal characterization and determination of nanoparticles essential oil content

Thermogravimetric analysis (TGA) was performed using a Shimadzu Model TGA-50 equipment. Samples were heated at a constant rate of 10°C min^-1^, over a temperature range of 25–900°C and under a nitrogen flow. The percentage of chitosan/essential oil was estimated from the fraction of derivative thermogravimetric analysis (dTG) area related to the last stage of mass loss, followed by an interpolation of the results (Eq 1):

$$\left(fCO-fEO\right)/\left(X_{CO}-0\right)=\left(fCN-fEO\right)/\left(100\%-0\right)$$ (1)

Where fCO is the mass fraction of the last peak of degradation minus the mass of the peak referring to water in chitosan nanoparticles loaded with essential oil thermogram, fEO is the mass fraction of the last peak of degradation minus the mass of the peak referring to water in essential oil thermogram and fCN is mass fraction of the last peak of degradation minus the mass of the peak referring to water in chitosan nanoparticle thermogram; and Xco is equal to the estimated chitosan fraction in the oil nanoparticle sample.

The essential oil (EO) encapsulation efficiency (EE) was estimated by Eq 2.

EE=(massofloadedEO/massofinitialEO)x100 (2)

#### Morphological and size characterizations

The morphology and particle size of the dried CO1:2 sample was determined by scanning electron microscopy (SEM). The lyophilized sample was covered with gold for 180 s using an Emitech apparatus (model K550) and observed using a Zeiss scanning electron microscope (Model DSM 962) at 15 kV. The nanoparticles were counted using the Image J program, with 400 observations, and the mean diameter with standard deviation was determined.

### Bioassays

To assess the toxic activity of the chitosan-encapsulated essential oil of *S*. *guianensis* in *Ae*. *aegypti* larvae, the larvicidal effects of essential oil-loaded chitosan nanoparticles at three different chitosan: essential oil ratios were monitored over time using different concentrations. For each bioassay, loaded essential oil chitosan nanoparticles were dispersed in 30 mL of distilled water. Every day, 25 third instar larvae of *Ae*. *aegypti* were added to each beaker, and after 24 h the dead larvae were counted, and all the larvae were removed. The procedure was repeated daily for 19 days or while there was larvicidal activity. The same procedure was repeated using only CN and only essential oil to identify the contribution of chitosan encapsulation. All experiments were performed in triplicate.

### Selectivity bioassays using aquatic non-target organisms

Here, we conducted two experimental sets in order to evaluate the potential selectivity of these loaded nanoparticles. In the first experimental set, we assessed the potential toxicity of the nanoparticles to adults of Guppy fishes (i.e., *Poecilia reticulata*). Briefly, Adults of *P*. *reticulata* (weight of 0.22 ± 0.13 g) collected in fish-farming installations at rural areas of Viçosa county (Viçosa, Minas Gerais state, Brazil, 20°45′ S, 42°52′ W) were brought to laboratory to an acclimation period. Taxonomists at the Department of Animal Biology (Federal University of Viçosa—UFV) identified the fishes. Once in laboratory, the fishes were maintained in glass aquaria (25 cm high x 25 cm width and 40 cm depth) containing dechlorinated tap water. These aquaria were kept under controlled conditions (i.e., 25 ± 3°C; 70 ± 5% relative humidity, 14:10 h light/dark regime and oxygen saturation of 8.1 ± 0.7 mg/mL). The toxicological studies were conducted using protocols previously described elsewhere [33–35] with slight modifications. We released two fishes into glass bows (2.5 L of capacity) containing 500 mL of a chitosan-encapsulated essential oil solution (chitosan:essential oil proportion of 1:2, which was the nanoparticle that exhibited the highest toxicity against mosquito larvae). The loaded essential oil chitosan nanoparticles concentration was of 0.83 mg/mL and the mortality was evaluated after 24 h. By using other fish groups, we also measured the residual effects of these solutions (always evaluating mortality at 24 h of exposure) during four consecutive days. The control treatment consisted of essential oil-free chitosan nanoparticles. For each treatment we used 20 replicates. The fishes were considered dead when were unable of swimming the distance equivalent of two-fold of their own body length.

Regarding the second experimental set, we evaluated the toxicity of the nanoparticles for embryos of zebrafish (i.e., *Danio rerio*). The *D*. *rerio* embryos were provided by the facility established at the Department of Genetics and Morphology, University of Brasília, Brazil. Prior obtaining the embryos, the zebrafish adults were kept in a ZebTEC (Tecniplast, Italy) recirculating system and maintained in aquariums with reverse osmosis and activated carbon filtered water. The temperature was maintained at 26.0 ± 1°C, ammonia < 0.01 mg/L, conductivity at 750 ± 50 mS/cm, pH at 7.5 ± 0.5 and dissolved oxygen equal to or above 95% saturation. Fish were raised in a 12:12 h (light:dark) photoperiod cycle. These conditions and water parameters were maintained in all the performed tests. Zebrafish eggs were obtained by breeding of fish in the Ispawn breeding system (Tecniplast). The day prior to breeding, males and females were sequentially added to the system and kept separated by a divider, in a proportion of two males for one female. Early in the morning, the divider was removed and the spawning platform was lifted to initiate the spawning. The eggs were collected immediately after natural mating, rinsed in water, and checked under a stereomicroscope (Stereoscopic Zoom Microscope e Stemi 2000, Zeiss, Germany). The unfertilized eggs (<20%) and those with cleavage irregularities or injuries were discarded.

The fish embryo toxicity test were based on the OECD guideline Protocol 236 [36]. The tests were performed using 10 eggs per concentration, divided in 6-well microplates. Five wells were filled-up with 10 mL of the test suspension and one well with water (internal plate control, as required in the OECD guideline). The test was initiated immediately after fertilization and it was continued for 96 h in a climate chamber (SL-24 Solab Científica, Brazil). Embryos and larvae were observed daily under a stereomicroscope.

### Statistical analysis

Mortality charts were plotted using SIGMA PLOT 11.0 software (Systat Software, Inc. San Jose CA, USA) and mortality data were subjected to Probit analysis using POLO PLUS statistical software (LeOra Software Berkeley, CA, USA).

## Results

### GC-FID and GC-MS analyses

The qualitative and quantitative composition of *S*. *guianensis* essential oil used in this study was analyzed using GC-FID and GC-MS (Table 2). These analyses revealed that the monoterpene *β*-myrcene (48.6%) and the sesquiterpene epicurzerenone (19.3%) were its major components.

**Table 2:** Chemical composition, concentrations (%) and Kovats index for *S*. *guianensis* essential oil determined by GC-FID and GC-MS.

Compound	Concentrations %	Ric*
Isoterpilonene	0.94	925
β-Myrcene	48.59	986
2-Undecanone	5.43	1271
ɣ-Elemene	7.39	1439
ɣ-Muurolene	0.67	1441
δ-Cadinene	0.57	1478
Germacrene D	9.93	1528
α-Cadinol	0.62	1592
Spathulenol	2.19	1547
Germacrene B	1.59	1613
Epicurzerenone	19.31	1611
Unidentified	2.77	-
Total identified	97.23	-

*Ric = Calculated retention index

### ATR-FTIR analysis

**Fig 1:**
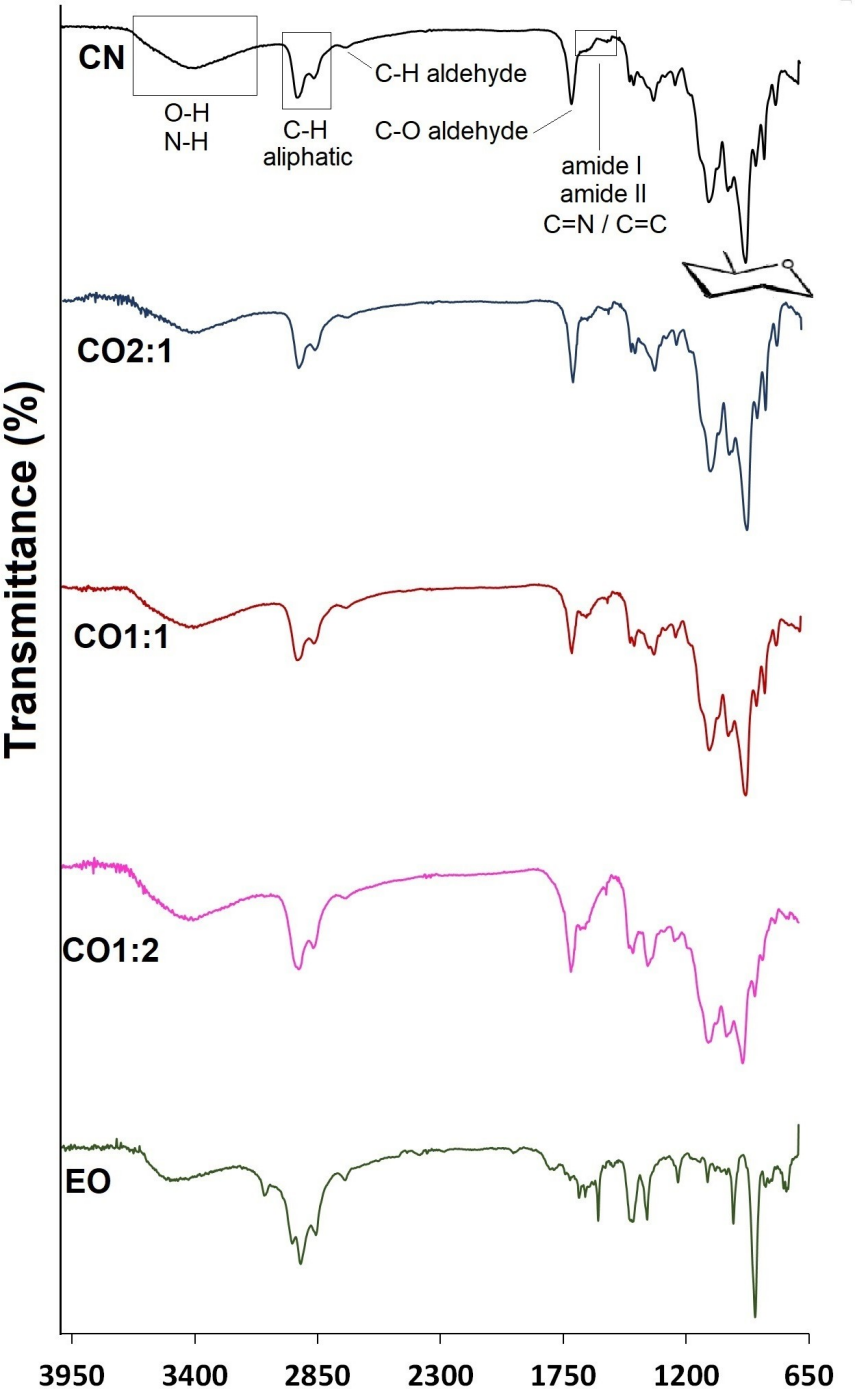
FTIR spectra of CN, CO2:1, CO1:1, CO1:2, and *S*. *guianensis* essential oil.

FTIR spectra of *S*. *guianensis* essential oil and nanoparticles are presented in Fig 1. The spectrum of chitosan nanoparticles presents the peaks related to the structure of the native polysaccharide [37–39] and the weak signals at ~1,647 cm^-1^ e ~1,560 cm^-1^, which can be attributed to peaks of amide I (C = O) and amide II (N–H bending), as well as the imine (C = N) and ethylenic groups (C = C) [40–42]. Peaks were also observed at 2,725 cm^-1^ and 1,718 cm^-1^, regions related to C–H and C = O bonds from an aldehyde, respectively [43].

The spectrum of the *S*. *guianensis* essential oil showed a broad band between 3,600–3,200 cm^-1^, characteristic of the hydroxyl functional group, and a band at 3,080 cm^-1^, attributed to the stretching vibration of C-H groups from olefins. Peaks at 2,960, 2,926, and 2,858 cm^-1^, as well as 1,447 and 1,377 cm^-1^, referring respectively to stretching vibrations and angular deformations of C-H bonds from aliphatic chains. The absorption peaks of double bonds from C = C present in unsaturated fatty acids were observed at 1,603 and 990–800 cm^-1^. The absorption at 1,799 cm^-1^ is characteristic of an ester (R-CO-OR) functional group; the absorption peaks at 1,236 and 1,110 cm^-1^ are related to ether (C-O-C) bonds, and the ones at 753 cm^-1^ are attributed to angular deformations of CH_2_ groups [43, 44].

The spectrum of the CO2:1 sample does not present significant differences from the CN sample. However, the increase in the proportion of EO used in the preparation of samples CO1:1 and CO1:2 induced a decrease in peak intensity at 940 cm^-1^ (pyranose rings) in relation to the other peaks.

### TGA

**Fig 2:**
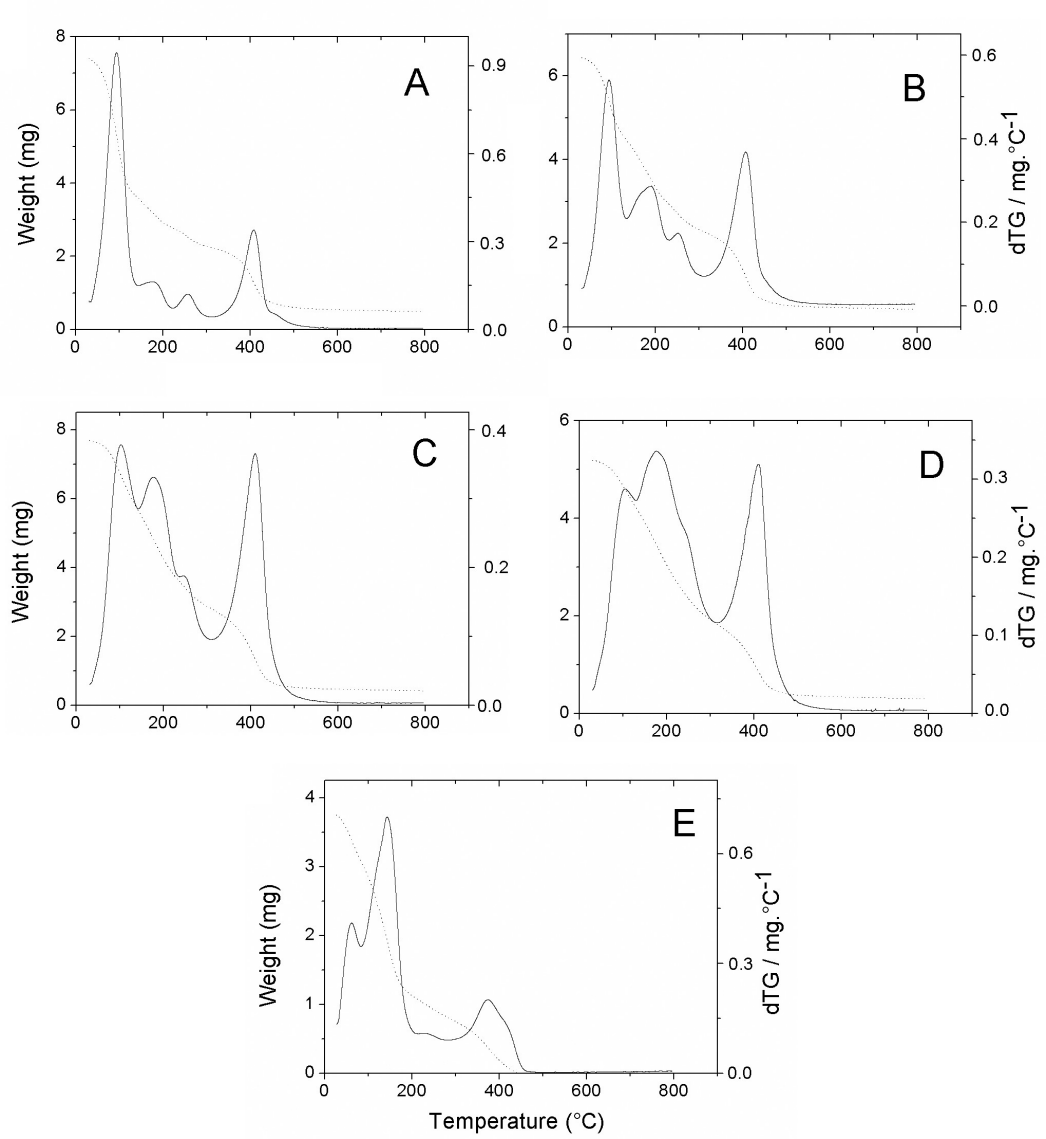
TG curves of (*A*) CN, (*B*) CO2:1, (*C*) CO1:1, (*D*) CO1:2, and (*E*) EO samples. (----) Residual weight and (^___^) dTG.

Fig 2 shows the thermogravimetric curves of the CN, CO2:1, CO1:1, CO1:2, and *S*. *guianensis* essential oil samples. The CN curve shows four stages of mass loss: the first step, from 25 to 140°C corresponds to the loss of adsorbed moisture and residual solvent, with a 50.26% mass loss. The second step occurred between 140 and 225°C with loss of 10.80% of total mass, due to degradation of the crosslinks formed by glutaraldehyde. The third and fourth ranges, between 225–310°C and 310–575°C, are related to the breaking of the main chain and decomposition of, respectively, 2-amino-2-deoxy-D-glucopyranose units and 2-acetamido-2-deoxy-D-glucopyranose units [45].

*S*. *guianensis* EO oil also shows four stages of mass loss. The first one between 33 and 90°C, corresponds to 18.15% of the sample weight and the second, from 90 to 213°C, represents a loss of 52.79%. The third and fourth stages occur between 213–320 and 320–450°C, respectively, and correspond to losses of 7.72% and 20.56%.

Although the nanoparticle CO2:1, with lower oil content, presented four stages of degradation with temperature ranges identical to those observed for CN, different mass losses were observed. The first step corresponds to the loss of 30.10%. The second, third and fourth stages presented mass losses of 23.30%, 9.60%, and 27.50%, respectively.

Samples CO1:1 and CO1:2 showed only three degradation steps with equal temperature ranges. In the first stage, 26.20% and 18.50% of the total masses were lost at a temperature of up to 140°C, respectively. The second stage from 140 to 310°C presented loss of 36.7% and 44.8%, respectively. In the third stage, with a range of 310 to 575°C, the mass losses were 30.8 and 28.3%.

The EO contents in the nanoparticles were estimated for samples CO2:1, CO1:1, and CO1:2 as 28%, 44%, and 58%, respectively. From those data, the encapsulation efficiency was calculated to be 84.8%, 88.0% and 87.0% for samples CO 2:1, CO1:1 and CO1:2, respectively.

### SEM analyses

**Fig 3:**
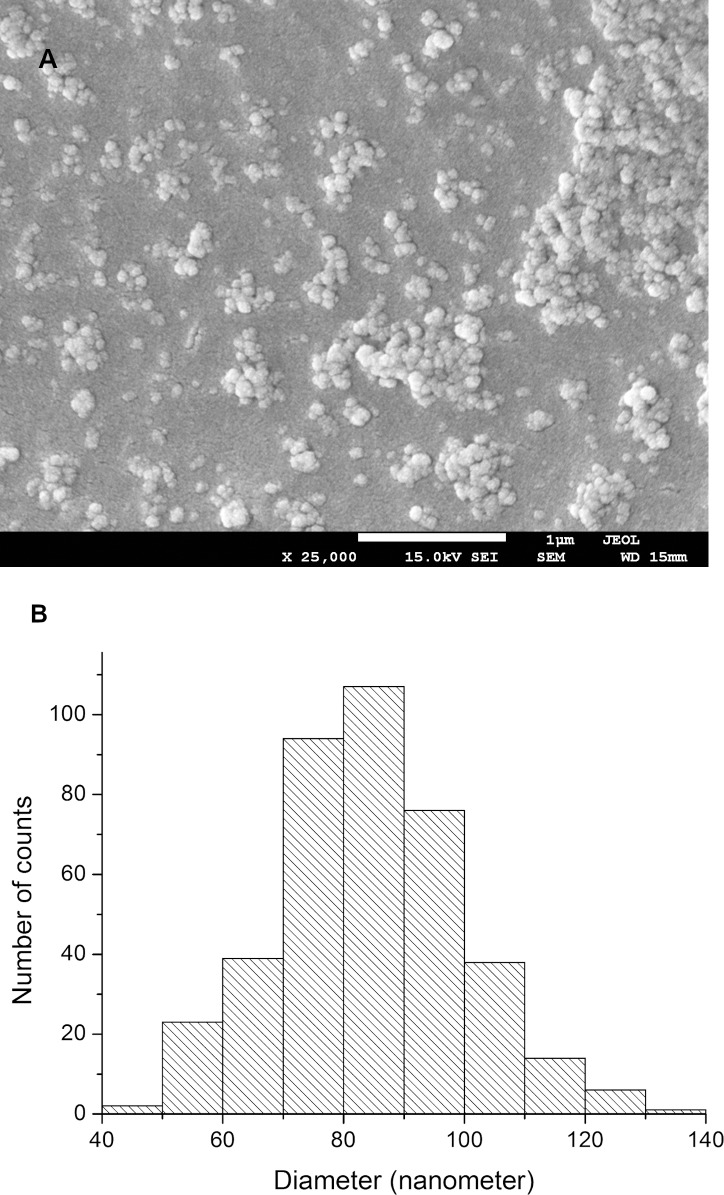
(a) SEM image of CO1:2 with an approximation of 25,000x and (b) the histogram obtained from 400 observations; Mean diameter = 82 nm, standard deviation = 15 nm and median = 84 nm.

Sample CO1:2 presented a regular appearance with a smoother particle surface (Fig 3A) and a mean diameter of 82 nm (Fig 3B).

### Bioassays

Bioassays were performed using varying concentrations of nanoparticles. Table 3 shows the essential oil content for each nanoparticle concentration used.

**Table 3:** The amounts of nanoparticles used in each bioassay, and the respective concentrations of *S*. *guianensis* essential oil.

	Concentration of essential oil of *S*. *guianensis* (mg/mL)		
**Mass (mg/30 mL)**	**CO2:1**	**CO1:1**	**CO1:2**
**25**	0.23	0.37	0.48
**50**	0.47	0.73	0.97
**75**	0.70	1.10	1.45
**100**	0.93	1.47	1.93
**150**	1.40	2.20	2.90
**200**	1.87	2.93	3.87

**Fig 4:**
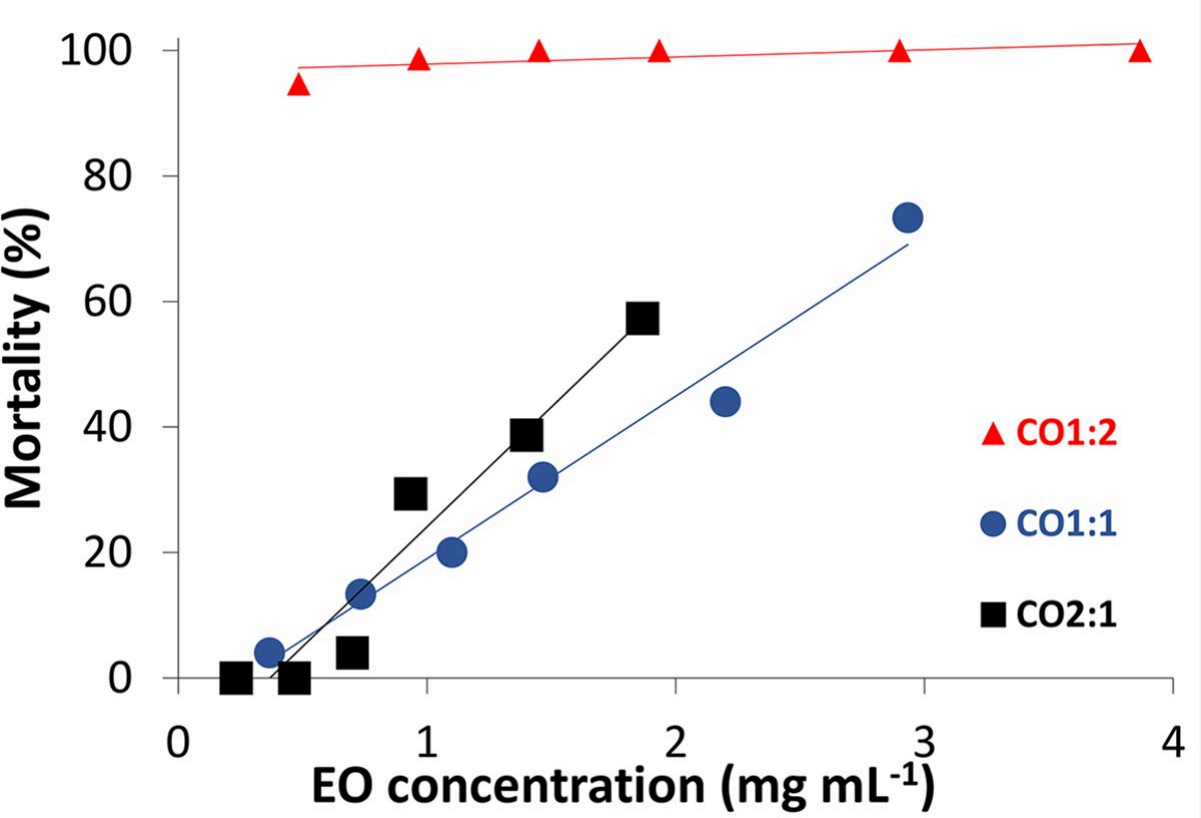
Mortality as a function of the net concentration of EO in the aqueous medium for different ratios between the EO and chitosan in the first 24 hours of exposure.

In this work, CN did not show any insecticidal activity against *Ae*. *aegypti* larvae. However, when using chitosan nanoparticles containing the essential oil, larvicidal activity was observed as a function of concentration and time. The data collected in the first 24 hours show the acute larvicidal activity of the nanoparticles with different loads (Fig 4). For sample CO2:1, whose load capacity is 28%, the lowest concentration from which larvicidal activity was observed was with the essential oil concentration of 0.70 mg mL^-1^. When using the CO1:1 nanoparticles, with a load capacity of 44%, larval death was observed with an EO concentration of 0.37 mg mL^-1^, lower than in the first case. In these two cases, mortality was below 10%. With the CO1:2 samples, bearing a load capacity of 58%, the lowest EO concentration used, 0.48 mg mL^-1^, resulted in 100% larval death in the first 24 hours of exposure.

**Fig 5:**
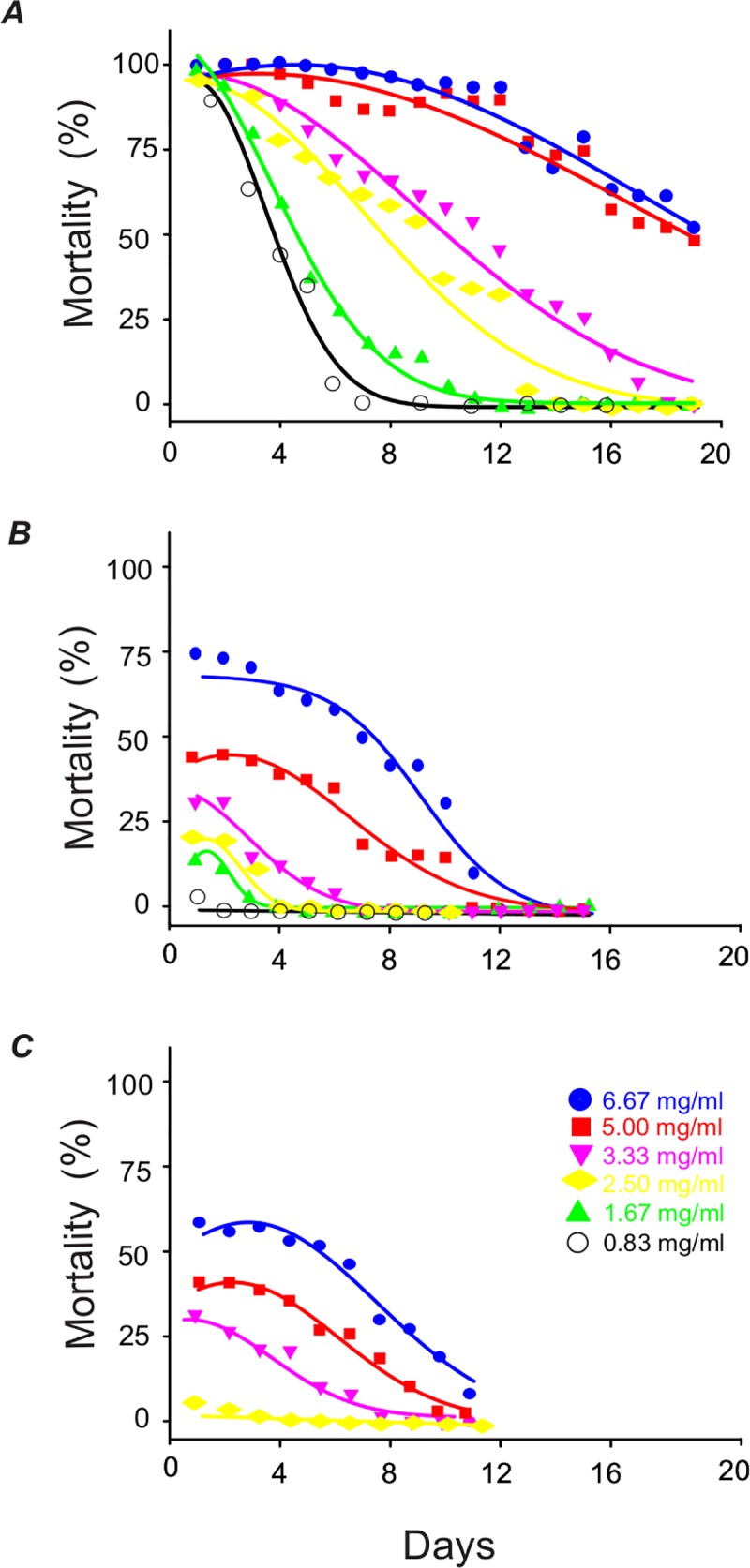
Residual larvicidal activity for different concentrations of chitosan nanoparticles containing *S*. *guianensis* essential oil against *Ae*. *aegypti* third instar larvae. (*A*) CO2:1, (*B*) CO1:1 and (*C*) CO1:2.

The residual toxicity assays showed that the persistence of larvicidal activity from chitosan nanoparticles loaded with essential oil depended on the dose and the chitosan:essential oil ratio used (Fig 5), and the results obtained for each concentration are statistically different (Table 4). While the mortality of *Ae*. *aegypti* larvae was below 60% for chitosan nanoparticles and EO at a ratio of 2:1 (CO2:1), and below 80% for nanoparticles at a ratio of 1:1 (CO1:1) when using the highest concentration of nanoparticles (6.67 mg mL^-1^), the nanoparticles at ratio of 1:2 (CO1:2) induced 100% mortality for more than 2 days at the lowest concentration (0.83 mg mL^-1^). The highest concentration of nanoparticles (6.67 mg mL^-1^) for the same chitosan:essential oil ratio (CO1:2) resulted in greater than 80% larval mortality for approximately two weeks From these results it was possible to estimate the mortality parameters for each chitosan:essential oil ratio, and for each concentration tested (Table 4).

**Table 4:** Mortality analysis and summary of non-linear regression analyses of Mortality parameters.

Ratio	Model	Concentration (mg/mL)	Estimated parameters (±SE)			*df*_error_	F	*P*	*R*^2^
			*a*	*b*	*y*_*0 or*_ *x*_*0*_				
**CO1:2**	y = a*exp (-0,5*((x-x_0_)/b)^2^)	0.83	0.96 (0.85–1.07)	2.57 (1.98–3.14)	0.74 (-0.18–1.65)	18	542.7	<0.0001	0.98
		1.67	1.07 (0.90–1.24)	3.9 (3.09–4.70)	-0.23 (-1.78–1.30)	18	787.5	<0.0001	0.99
		2.5	0.96 (0.83–1.09)	6.34 (4.67–8.02)	1.00 (-1.6–3.6)	18	201.0	<0.0001	0.96
		3.33	0.98 (0.88–1.09)	8.24 (6.59–9.89)	0.67 (-1.9–3.3)	18	285.5	<0.0001	0.97
		5.00	0.97 (0.93–1.02)	13.50 (10.42–16.73)	3.32 (0.39–6.24)	18	86.4	<0.0001	0.91
		6.67	1.00 (0.9–1.0)	12.80 (10.9- -14.7)	4.55 (3.03–6.07)	18	153.6	<0.0001	0.95
**CO1:1**	y = ax+ y_0_	0.83	-0.001 (-0.002–0.0002)	-	0.01 (-0.0007–0.02)	14	3.0	0.0057	0.43
	y = a/(1+exp (-(x-x_0_)/b))	1.67	0.16 (0.15–0.16)	0.82 (0.80–0.83)	1.50 (1.49–1.50)	14	457.7	<0.0001	0.99
		2.5	0.20 (0.18–0.22)	1.24 (1.00–1.48)	1.37 (1.12–1.62)	14	417.2	<0.0001	0.98
		3.33	0.35 (0.25–0.44)	2.79 (1.77–3.82)	0.01 (-1.94–1.97)	14	299.9	<0.0001	0.98
		5.00	0.44 (0.40–0.48)	4.26 (3.27- -5.25)	2.20 (0.97–3.44)	14	173.2	<0.0001	0.96
		6.67	0.69 (0.62–0.76)	-1.58 (-2.14- -1.01)	8.85 (8.17–9.54)	14	203.2	<0.0001	0.97
**CO2:1**	y = ax+ y_0_	2.50	-0.003(-0.005- -0.0001)	-	0.02 (0.00–0.036)	10	5.7	0.04	0.64
	y = a*exp (-0,5*((x-x_0_)/b)^2^)	3.33	0.28 (0.22–0.33)	2.79 (1.52–4.06)	1.13 (-0.67–2.94)	10	83.0	<0.0001	0.95
		5.00	0.39 (0.36–0.42)	3.50 (2.82–4.18)	2.14 (1.34–2.94)	10	182.1	<0.0001	0.98
		6.67	0.57 (0.53–0.61)	4.23 (3.39–5.08)	2.48 (1.57–3.39)	10	121.3	<0.0001	0.97

### Selectivity bioassays using aquatic non-target organisms

The exposure of adults of *P*. *reticulata* fishes to loaded essential oil chitosan nanoparticles (0.83 mg/mL) inflicted in a mortality rate of less than 30% in the first 24 h. Such fish mortalities significantly decreased over time with almost no detectable mortality after 72 h of exposure (S1A Fig). A similar mortality was observed for *D*. *rerio* embryos when exposed to a concentration of 0.45 mg/mL throughout the observation period (S1B Fig). No changes were observed in embryos treated with concentrations less than 0.10 mg/mL.

## Discussion

Eco-friendly control tools are needed for the management of insect pests of agricultural and medical relevance. Plant-extracted essential oils and their major components are seen as potential sources for alternatives to synthetic insecticides in the control strategies for insect pests, as they are considered safe for the environment and human health [13, 46]. However, their use on a wide-scale presents challenges due to their volatility and susceptibility to degradation under environmental conditions of high temperatures, UV light, and oxidation. Here, we tested the encapsulation of essential oil extracted from Negramina, *S*. *guianensis*, plants in chitosan nanoparticles crosslinked with glutaraldehyde as a feasible and efficient approach to increase physical stability and to protect from degradation, as well as to modulate its release for better control of larva of the mosquito *Ae*. *aegypti*. Furthermore, we also demonstrated the selectivity of this mosquito control tool against zebrafish (i.e., *D*. *rerio*) embryos and insectivorous adult fishes (i.e., *P*. *reticulata*).

Our chromatographic analyses of the *S*. *guianensis* essential oil revealed that the monoterpene β-myrcene (48.6%), the sesquiterpenes epicurzerenone (19.3%), germacrene D (9.9%), and ɣ-elemene (7.4%), and the non-terpenic acyclic ketone 2-undecanone (5.43%) were its major components. While this chemical profile is different from other profiles of the essential oil obtained from *S*. *guianensis* grown in other Brazilian regions and described in previous investigations [47–51], it is in concordance with our recent studies with *S*. *guianensis* essential oil extracted from stems and leaves [14, 52].

The thermogravimetric curve derived from *S*. *guianensis* essential oil also presented some differences from the one previously published, with the first and second stages of mass loss corresponding to a loss of 70%, higher than that previously observed [44]. Quantitative and qualitative variations in chemical composition of essential oils are attributed to various factors, e.g., ecotype, phenophase, temperature, relative humidity, photoperiod, irradiance, genotype, extraction method, and agronomic conditions [53, 54].

Recent investigations of *S*. *guianensis* essential oil have shown its potential in controlling and repelling mosquitos [14], cattle ticks [49], and wax moths [15]. Such activity is assumed to be related to its major components. Indeed, β-myrcene [14, 55], germacrene D [56, 57], ɣ-elemene [58, 59], and 2-undecanone [60] have been shown to exert insecticidal or repellent activity across several insect and mite species, including mosquito species. Although the mode of action of *S*. *guianensis* essential oil was not addressed in detail here, previous *in vitro* studies reported necrotic and apoptotic cell death both in mosquito C6/36 cell lines [14] and lepidopteran cultured cells [61]. Such toxic effects might derive from the actions of terpenes (e.g., the monoterpene *β*-myrcene) present in the essential oil. Essential oils with high monoterpene contents have been shown to exert their action on octopamine, tyramine, GABA, and TRP channels [62–64]. Moreover, previous investigations [61] have also shown that the *S*. *guianensi*s essential oil did not alter the viability of a human monocytic cell line (TPH1), suggesting the existence of differential target susceptibilities between vertebrate and insect cells.

Although essential oils have been intensely investigated for their biological activities, their use is still limited because of their high volatility, rapid oxidation, and degradation on contact with air, as well as their low solubility in water [28, 65, 66]. Encapsulation and nanoencapsulation of essential oils using polymers have drawn focus as some of the most promising techniques to overcome such limitations [67, 68].

Depending on their applicability, safety, biocompatibility, cost, and availability, various polymers have been tested for the encapsulation of essential oils, including gums, alginates, chitin and polyesters (e.g., polyethylene glycol, poly-ε-caprolactone) as well as starch and its derivates (dextrins, maltodextrins, cyclodextrins) [69]. In this broad range of polymers, chitosan (a deacetylated derivative of chitin) stands out as non-toxic, biocompatible, biodegradable, and mainly produced from renewable sources [20]. Furthermore, in recent years, several publications have described nanometric systems based on chitosan to encapsulate essential oils.

However, most of these publications only study the structural characterization and physicochemical properties of these oil-loaded nanoparticles, and very few articles describe the applicability of chitosan nanoparticles as adjuvants of compounds with larvicidal activity [26–28] and none of them verified whether chitosan nanoparticles containing the essential oil may pose any risk to non-target organisms. Thus, we prepared chitosan nanoparticles using glutaraldehyde as a crosslinking agent and incorporated the essential oil into these nanoparticles during their preparation with the objective of verifying whether the encapsulation of the essential oil in these nanoparticles increases its stability in an aqueous medium and allows its use as a larvicide.

The crosslinking of chitosan with glutaraldehyde is well-documented in the literature and, to confirm the formation of crosslinks, the CN sample, which does not contain the essential oil, was characterized by FTIR and TGA. Although the signals at 1,647 cm^-1^ and 1,560 cm^-1^ in the CN spectrum can both be attributed to amide I (C = O) and amide II (N–H bending) from partially deacetylated chitosan, as well as the imine (C = N) and ethylenic groups (C = C) formed by the reaction between the amino groups of the polymer with the aldehyde groups of glutaraldehyde, the thermogravimetric curve of the CN sample shows four stages of mass loss. These four stages are characteristic of glutaraldehyde cross-linked chitosan. The 10% mass loss observed between 140 and 225°C is probably due to degradation of the crosslinks formed by glutaraldehyde [38], confirming the formation of cross-links, although not all glutaraldehyde was consumed in this reaction, as indicated by the peaks in the 2,725 cm^-1^ and 1,718 cm^-1^, regions related to C–H and C = O bonds from aldehyde.

The peaks at 1,647 cm^-1^ and 1,560 cm^-1^ also appear in the FTIR spectra of samples CO2:1, CO1:1, and CO1:2, prepared with essential oil, indicating that essential oil presence does not interfere with the crosslink formation. The FTIR spectrum of sample CO2:1 does not have significant differences compared with that of CN, probably due to the lower proportion of EO used in its preparation. The only differences observed in the TG curve was a smaller loss of mass in the first stage compared sample CN, probably due to a smaller amount of water adsorbed in these particles, due to its more hydrophobic character conferred by the presence of the EO.

The FTIR spectra of CO1:1 and CO1:2 showed a decrease in peaks from pyranose rings compared with the other peaks, indicating an increase of the other functional groups in relation to the main chitosan chain. This decrease was also observed with other chitosan nanoparticles loaded with essential oil [32]. As expected, the mass loss in TGA curves due to loss of moisture decreased as the percentage of oil increased in the nanoparticles. The second and third stages of CN and CO2:1 were masked, probably due to the concomitant degradation of the essential oil, which presents a considerable loss of mass in this range.

It is possible to determine the load capacity of this type of nanoparticles using TGA by discounting the water mass of the materials and using the data referring to the area of the derivative (dTG) [22, 23, 70]. In our study, the load capacity of the obtained nanoparticles ranged from 28 to 58%, while the encapsulation efficiency was higher than 84%. Such values suggest that the crosslinking with glutaraldehyde is an interesting strategy for the encapsulation of essential oils. Indeed, essential oil load capacity and encapsulation efficiency values found in the literature, calculated from the TGA for chitosan nanoparticles crosslinked with tripolyphosphate, and using the same proportions of chitosan:essential oil are lower than those determined in this study [32].

After characterizing the nanoparticles, we evaluated their larvicidal effect in the ratios that are shown in Table 1. The most efficient chitosan:essential oil ratio was 1:2, as it achieved complete mosquito larvae mortality, and the high activity of loaded particles was maintained for up to two weeks. To verify if this result is due only to the higher medium oil concentration, or is also affected by the chitosan:essential oil ratio of each sample, the mortality caused by the same amount of oil in a 24-hour interval was compared. These results showed that the nanoparticle load capacity also affects the larvicidal activity of essential oil. Thus, by increasing the nanoparticle load, the amount of oil applied in the medium can be reduced.

Although the insecticidal activity of chitosan has been observed against wax moth larvae [71], studies with pure chitosan nanoparticles showed no effect on larvae of *Anopheles gambiae* [72] and *Ae*. *aegypti* [73]. Our tests are in line with these results, where CN alone also did not show any larvicidal activity against *Ae*. *aegypti* larvae. We also observed that the essential oil, when applied alone and without the use of any solubilizer could not reach the larvae, indicating that the mortality observed in the bioassays is due to the ability of the chitosan as compatibilizer of the oil with water and to protect it from changes that affect its toxicity.

The high efficacy of the encapsulated essential oil could be due to various parameters. First of all, the increase of the essential oil load into nanoparticles would increase the amount of essential oil adsorbed on the nanoparticle surface, with a subsequent increase of the burst effect. Secondly, better penetration of the essential oil-loaded nanoparticles in the larval bodies may be facilitated by their nanometric size, which for the CO1:2 sample was 82 nm, close to that found by other authors for SEM of different dried chitosan-based nanoparticles loaded with essential oil [31, 74]. This small size may also enhance the contact and action of the active components of the essential oil at their action target [28, 75]. Similarly enhanced penetration into cells was previously reported for nano-encapsulated insecticides [76–78].

Furthermore, because the larvae are recyclers and feed on particulate matter present in the medium, chitosan nanoparticles can act through a “Trojan horse” mechanism, following ingestion by the larvae. The higher the essential oil load on nanoparticles, the smaller the number of ingested nanoparticles required to have a larvicidal effect, as observed in Fig 4. After ingestion, the nanoparticles may release their toxic content into the digestive tract of the larvae, or be absorbed by endocytosis [72, 79]. The cationic nature of the chitosan probably also contributes to the enhancement of the essential oil toxicity, since positively charged nanoparticles are efficiently internalized by cells and escape from lysosomes to the cytosol, as observed with poly(ethyleneglycol) nanoparticles in tests with *A*. *gambiae* [79].

The surrounding layer formed by chitosan in nanoparticles could help protect the essential oil from the detoxifying enzymes inside the insect body [77, 78]. This protective role played by the encapsulating polymer probably supports the prolonged larvicidal effects observed in our study, as it enabled the protection of the insecticide from degradation and volatilization [80–82]. Other authors have also observed that the encapsulation of essential oils in chitosan nanoparticles increase their half-lives [83–85].

After we verified the positive effect of the encapsulation of essential oil on chitosan nanoparticles on its larvicidal activity, we evaluated whether the nanoparticles obtained could have some toxic effect on Guppy, *P*. *reticulata*, fishes that is not only well known to prey upon mosquito larvae but is also one of the species recommended by the OECD for the performance of acute toxicity tests on fish [86]. The evaluation of fish toxicity is useful in the assessment and management of ecological risk, because fish serves as an important bioindicator for aquatic systems. The test was done with the CO 2:1 sample, which presented the highest toxicity to the mosquito larvae. It was possible to observe that, even in a concentration 2.3 times higher than the concentration suggested as limit in the acute toxicity tests on fish, the mortality was less than 15% (S1A Fig). This toxicity is lower than that observed with pyrethroids for fish and other aquatic organisms, for example [87].

Furthermore, as the presence of these compounds in an aquatic system can directly affect not only mosquito larvae but also other non-target organisms (e.g., predators preying upon mosquito larvae), it is of crucial importance to characterize the potential detrimental or sublethal effects of such nanoparticles on the behavior and reproduction of such non-target organisms. Indeed, recent investigations have shown that sublethal exposure of insects to eco-friendly insecticides can cause not only detrimental [61, 88–92] but also stimulatory [93–95] responses that either increase or compromise the efficacy of these compounds. Then, after the toxicity evaluation of the chitosan nanoparticles containing the essential oil of *S*. *guianensis* on a predatory species of *Ae*. *aegypti*, we evaluated its toxicity on *D*. *rerio* embryos. Although essential oils are often considered to be safer, a similar experiment with the thyme essential oil showed that it was able to cause 100% of embryo mortality at the concentration of 0.05 mg/mL at 72 hours post fertilization (hpf) [96]. In our experiment, 100% mortality occurred when a 0.9 mg/mL concentration was used and it is possible to observe that the mortality observed for the embryos did not exceed 30% even using a concentration 4.5 times higher than the recommended for the limit test (S1B Fig).

## Conclusions

Chitosan nanoparticles chemically crosslinked with glutaraldehyde and loaded with *S*. *guianensis* essential oil were successfully prepared and their larvicidal activity was tested against *Ae*. *aegypti* larvae. All chitosan:essential oil ratios evaluated had better larvicidal activity than just the oil without adjuvants. It was also observed that the chitosan:essential oil ratio affects larvicidal activity. The higher the proportion of essential oil in the nanoparticle, the higher the larvicidal activity, even with low net essential oil concentrations in the medium. The inclusion of the essential oil in chitosan nanoparticles also prolonged its larvicidal effect, indicating that these nanoparticles are suitable for preserving the essential oil properties in an aqueous medium and protects from degradation.

Although not addressed in detail in this investigation, the studies with non-target organisms indicate that the essential oil of *S*. *guianensis* encapsulated in chitosan nanoparticles caused a low mortality in these organisms, not causing death or alterations in zebrafish embryos in the indicated concentration for the execution of the limit test, 0.1 mg/mL.

The results obtained in this study corroborate with other laboratory tests regarding the efficiency of botanical insecticides, however, considering that the studies presented here were performed under conditions different from those found in the field (30 mL used volume) it will be necessary that these products be evaluated in the field in order to develop commercial mosquito larvicides of botanical origin. Further experiment analyzing the complex dynamics of natural water matrices where mosquito larvae live will allow for better testing the stability of chitosan encapsulated particles of *S*. *guianensis* essential oil as well as their release kinetics under realistic field conditions. Thus, such investigations will help in understanding the full potential of nano-encapsulating *S*. *guianensis* essential oil as an alternative to chemical control of mosquitoes.

## Supporting information

S1 Fig Selectivity against non-target fishes. (A) adults of *Poecilia reticulata*. (B) embryos of *Danio rerio*.
(PDF)
