# Supplementary material for: Prolonged mosquitocidal activity of Siparuna guianensis essential oil encapsulated in chitosan nanoparticles
Source: PLoS Negl Trop Dis. 2019 Aug 9;13(8):e0007624. doi: 10.1371/journal.pntd.0007624 (PMC6703692; doi:10.1371/journal.pntd.0007624)
Supplement: S1 Fig — (A) adults of Poecilia reticulata. (B) embryos of Danio rerio. (PDF) [file pntd.0007624.s001.pdf]

selectivity against non-target fishes

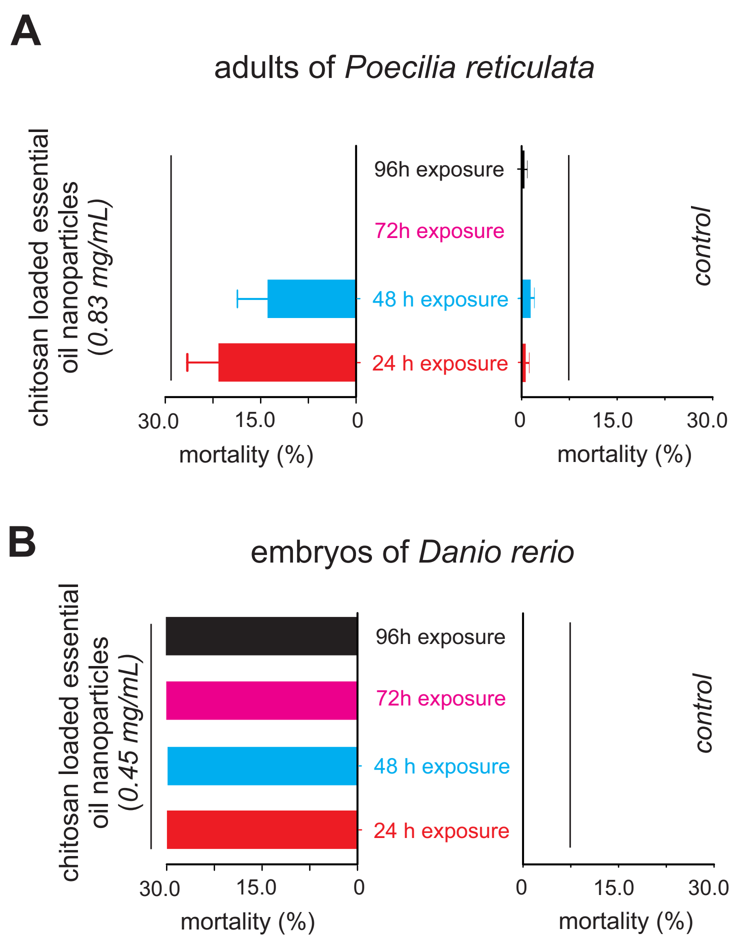

Supplementar Figure 1
